# From gaps to progress: five-year global advances in the diagnosis of pediatric tuberculosis

**DOI:** 10.3389/fped.2026.1778870

**Published:** 2026-03-17

**Authors:** Yang Yang, Dan Ye, Zhentao Fei, Ping Liu, Huarui Liu, Xuhui Liu, Lu Xia

**Affiliations:** Department of Tuberculosis, Shanghai Public Health Clinical Center, Fudan University, Shanghai, China

**Keywords:** artificial intelligence, host-response biomarkers, molecular diagnostics, pediatric tuberculosis, point-of-care testing

## Abstract

**Background:**

Pediatric tuberculosis (TB) remains a major global health challenge, characterized by age-specific difficulties especially in diagnosis compared to adults. This review summarizes key advances in pediatric TB diagnostics over the past five years.

**Methods:**

Literature was retrieved from PubMed/MEDLINE, Embase, Web of Science, Scopus, the Cochrane Library, and Google Scholar. Priority was given to multi-center pediatric cohort studies, diagnostic accuracy studies, implementation studies, and evidence that informed international guidelines.

**Advances:**

In diagnosis, the field is shifting from reliance on conventional bacteriology toward a multidimensional approach integrating molecular diagnostics, host biomarkers, and artificial intelligence (AI)-assisted imaging. This approach addresses challenges arising from the paucibacillary nature of pediatric TB and difficulties in specimen collection. Recent advances include expanded use of rapid molecular assays on non-sputum specimens, the development of host-response biomarkers primarily for triage and risk stratification, and emerging but still limited applications of AI-assisted chest radiography for screening in pediatric populations.

**Conclusion:**

Although pediatric TB care has moved from an adult-centric evidence paradigm to an increasingly child-specific evidence base, substantial gaps persist between diagnostic evidence and real-world implementation, especially in high-burden, resource-constrained settings. Future efforts should prioritize scalable, child-centered diagnostic strategies that can be integrated into decentralized care pathways, with particular attention to infants and other vulnerable groups. Strengthening diagnostic access and implementation will be critical to advancing global End TB goals for children.

## 1 Introduction

Tuberculosis (TB) remains one of the most challenging infectious diseases globally despite decades of control efforts, even though it is preventable and curable. Children under 15 years are particularly vulnerable and face disproportionate challenges in TB diagnosis compared with adults. Among children with TB, 174,300 died, accounting for 14.2% of all TB deaths in 2024 (1). Seventy-four percent of these deaths were among children under five years, who are uniquely vulnerable to rapid progression to severe TB disease (1, 2). Furthermore, an estimated 52% of children under five years are undiagnosed, untreated or unreported, and 96% of the children who died from TB did not receive TB treatment (2).

Underdiagnosis of TB in children results from several key challenges. First, clinical presentations are nonspecific and overlap with other common childhood illnesses, often leading to misdiagnosis or delay. Second, the typically low bacillary load in pediatric TB often falls below the detection threshold of current bacteriological methods (3, 4). Compounding these biological limitations is the persistent lack of sensitive, child-friendly diagnostic tools that can be deployed at or near the point of care (POC). Additionally, difficulty obtaining high-quality respiratory specimens, especially from young children, often forces the use of invasive methods like sputum induction or gastric aspiration (5, 6). Many children with TB are diagnosed clinically without bacteriological confirmation, especially a common practice in resource-limited settings—a practice that increases the risk of both overdiagnosis and underdiagnosis (7). These diagnostic gaps represent a critical bottleneck in the pediatric TB care cascade, directly contributing to delayed treatment initiation, missed cases, and preventable mortality. Addressing them requires diagnostic strategies that account for the biological, clinical, and health-system constraints unique to children.

In recent years, substantial progress has been made in pediatric TB diagnostics, including advances in rapid molecular assays applicable to non-sputum specimens, host-response–based biomarkers for triage and risk stratification, and artificial intelligence-assisted imaging tools to support screening and diagnostic decision-making. These innovations aim to improve microbiological confirmation while reducing reliance on invasive sampling and highly specialized laboratory infrastructure. Accordingly, this review focuses on global advances (2020–2025) in the diagnosis of pediatric TB. We synthesize recent evidence on molecular diagnostics, host biomarkers, and AI-based approaches, and critically examine their performance characteristics, implementation feasibility, and potential roles within child-centered diagnostic algorithms, particularly in high-burden, resource-constrained settings.

## 2 Methods

This review summarizes advances in pediatric TB diagnostics from December 1, 2020 to December 31, 2025, with selective inclusion of pivotal earlier trials and international guidelines to contextualize recent developments. PubMed/MEDLINE served as the primary database for study identification and screening, with additional literature identified from Web of Science, Embase, Scopus, the Cochrane Library, and Google Scholar. Searches were conducted between December 1, 2020 and December 31, 2025. Search strategies combined controlled vocabulary and free-text terms using Boolean operators, including [“tuberculosis” AND (“child” OR “childhood” OR “adolescent” OR “pediatric”)] paired with (“diagnosis” OR “rapid molecular diagnostics” OR “molecular testing” OR “biomarkers” OR “host response” OR “artificial intelligence” OR “computer-aided detection”). Terms related to treatment, drug resistance, and TB preventive treatment were intentionally excluded to align with the diagnostic focus of this review. Search strategies were adapted to the syntax and indexing terms of each database. Titles and abstracts were screened for relevance, followed by full-text review of eligible articles. This review was narrative rather than systematic, and no formal risk-of-bias assessment or quantitative synthesis was performed. A PRISMA-informed approach was used to enhance transparency in study identification and selection, without applying formal systematic review criteria (Supplementary Figure S1). In addition, reference lists of key articles and guidelines were manually screened to identify seminal studies published prior to the predefined search window when necessary to contextualize current advances.

Priority was given to prospective cohort studies, randomized controlled trials (RCTs), individual participant data (IPD) meta-analyses, and WHO-convened evidence syntheses that included pediatric-specific data or subgroup analyses. Adult studies were included only when they constituted the primary or foundational evidence base for diagnostic tools subsequently applied to children (e.g., rapid molecular assays, host-response signatures, or AI-based imaging platforms), and where pediatric validation data were limited or emerging.

For diagnostics, the review focused on WHO-recommended rapid molecular diagnostics, emerging host-response and biomarker assays, and artificial intelligence-assisted imaging tools with available pediatric data or clear relevance to pediatric implementation. Studies without direct or inferable applicability to pediatric TB diagnosis were excluded.

This review was not designed as a formal systematic review, and no quantitative meta-analysis or formal risk-of-bias assessment was performed. A pragmatic, PRISMA-informed approach was adopted to enhance transparency while prioritizing high-quality, multi-center studies and guideline-influencing evidence relevant to pediatric TB diagnosis. Evidence gaps, reliance on adult extrapolation, and remaining research priorities in pediatric TB diagnostics are explicitly highlighted.

## 3 A new diagnostic landscape: molecular nucleic acid amplification tests (NAATs), host-response tools, AI, immunodiagnostics

Bacteriological confirmation is achieved in only 7%–40% of pediatric patients with pulmonary TB (8–11), driving efforts to improve sensitivity of molecular assays and expand access to non-sputum specimens (stool, urine) and host-response diagnostic alternatives. In this section, we review WHO-recommended and emerging diagnostic tools for pediatric tuberculosis, including rapid molecular assays (Xpert MTB/RIF and Xpert MTB/RIF Ultra, Truenat), low-complexity nucleic acid amplification tests, such as TB- Loop-mediated isothermal amplification (LAMP), molecular drug-resistance assays (line probe assays), and selected emerging platforms such as CRISPR-based diagnostics and targeted next-generation sequencing. To facilitate comparison across diagnostic modalities and specimen types, the key performance characteristics of these assays are summarized in Table 1, with detailed discussion of individual platforms provided in the subsections below. Detailed study-level performance characteristics are provided in Supplementary Tables S1–S6.

**Table 1:** Performance, specimen types, and WHO-recommended roles of molecular and emerging diagnostic tests for pediatric tuberculosis.

Diagnostic category	Assay/platform	Specimen types (pediatric)	Reference standard	Diagnostic performance (range)	WHO recommendation/role	Key notes	Source
mWRDs	Xpert MTB/RIF	Induced sputum, GA, stool, NPA	Culture/CRS	Sens: ∼45%–73%; Spec: >98%	WHO-recommended initial mWRD	Reduced sensitivity in paucibacillary TB	Supplementary Table 1
	Xpert MTB/RIF Ultra	Induced sputum, GA, stool, NPA, CSF	Culture/CRS	Sens: ∼44%–80%; Spec: ∼94%–98%	WHO-preferred initial mWRD	Improved sensitivity; trace calls	Supplementary Table 1
	Truenat MTB/MTB Plus + MTB-RIF Dx	GA, induced sputum, BALF	MRS/culture/CRS	Sens: ∼57%–59%; Spec: ∼88%–92%	WHO-recommended initial mWRD (near-POC)	Lower infrastructure; reflex RI*F* testing	Supplementary Table 2
Low-complexity NAATs	TB-LAMP	GA, NPA, BALF	Culture/CRS	Sens: ∼50%–78%; Spec: ∼67%–100%	WHO-endorsed alternative NAAT in specific settings (not first-line)	High specimen-dependence; not first-line	Supplementary Table 3
Molecular drug-resistance assays	Line probe assays (MTBDRplus/sl)	GA, sputum (smear+/culture+)	Phenotypic DST/CRS	Sens/Spec for RIF/INH/MDR: ∼90%–99%^a^	WHO-endorsed for genotypic DST	Limited data in smear-negative children	Supplementary Table 4
Emerging molecular diagnostics	CRISPR-Cas assays	cfDNA, induced sputum, stool	CRS/culture/MRS	Sens: ∼83%–100%; Spec: ∼94%–95%	Research/proof-of-concept	Promising in paucibacillary & EPTB	Supplementary Table 5
Sequencing-based diagnostics	tNGS	Respiratory & extrapulmonary	CRS/phenotypic DST	Sens: ∼18%–94%; Spec: ∼87%–98%	WHO-endorsed for comprehensive DST^b^	Useful in culture-negative/DR-TB	Supplementary Table 6
	WGS	Culture isolates	NA (surveillance)	NA	Surveillance/epidemiology	Not for frontline diagnosis	

mWRDs, WHO recommends rapid molecular diagnostics; GA, gastric aspirate; NPA, nasopharyngeal aspirate; BALF, bronchoalveolar lavage fluid; CRS, composite reference standard; MDR, multidrug-resistant; RIF, rifampicin; INH, Isoniazid; MRS, microbiological reference standard; DST, drug susceptibility testing; EPTB, extrapulmonary tuberculosis; tNGS, targeted next-generation sequencing; WGS, whole-genome sequencing; DR-TB, drug-resistant tuberculosis.

^a^ Performance estimates for line probe assays are primarily derived from adult or mixed populations and from smear-positive or culture-positive specimens. In children, particularly those with paucibacillary or smear-negative disease, sensitivity is substantially lower, and data remain limited.

^b^ WHO endorsement for sequencing-based DST applies primarily to reference laboratories and programmatic use rather than frontline pediatric diagnosis.

### 3.1 Rapid molecular diagnostics

Microbiological confirmation of tuberculosis in children remains challenging because pediatric disease is typically paucibacillary and respiratory specimens are often difficult to obtain (3). Smear microscopy has extremely limited sensitivity in pediatric TB, while mycobacterial culture—although considered a reference standard—is constrained by long turnaround times and the need for specialized laboratory infrastructure, limiting its utility for timely decision-making in many settings (3, 12). These limitations support the use of rapid molecular diagnostics as initial tests in children with presumptive TB (12, 13). To address these challenges, the World Health Organization (WHO) recommends rapid molecular diagnostics (mWRDs) as initial diagnostic tests for people with presumptive TB, including children (12, 14). These NAATs provide substantially higher sensitivity than smear microscopy and enable rapid detection of rifampicin resistance to guide early management (12, 14). Global guidelines therefore recommend replacing smear microscopy with WHO-endorsed rapid molecular diagnostics as initial tests for presumptive TB in children, given the extremely low sensitivity of smear microscopy in young children, particularly among those living with HIV. WHO-endorsed mWRDs for initial testing include cartridge-based assays such as Xpert MTB/RIF and Xpert MTB/RIF Ultra, detecting Mycobacterium tuberculosis (M.tb) DNA and rifampicin resistance, as well as low-complexity near-POC platforms such as Truenat, with test selection guided by specimen type, setting, and resource availability (12, 13).

In this subsection, we review the performance, clinical utility, and implementation considerations of WHO-recommended molecular diagnostics in children, followed by emerging molecular and sequencing-based tools that may further improve diagnostic yield and resistance detection.

#### 3.1.1 Xpert/RIF platform: establishment of the ultra era and performance enhancement

Xpert MTB/RIF and MTB/RIF Ultra are the most widely used cartridge-based NAATs in pediatric TB. Xpert MTB/RIF Ultra has shown higher sensitivity than its predecessor, especially for low-bacillary and non-invasive specimens like stool (15–19), by incorporating multi-copy targets (IS6110, IS1081) and expanding the PCR chamber and lowering the limit of detection (LOD) from 131 CFU/mL to 15.6 CFU/mL (20) (Table 1). A South African study on pediatric induced sputum showed Ultra increased detection of microbiologically confirmed patients with pulmonary TB from 63.2% to 73.7%, compared to Xpert (15). Multi-national pediatric cohorts confirm Ultra's superiority in non-invasive specimens. Notably, the high sensitivity of Ultra involves a trade-off with specificity, raising the potential for false positivity, especially for results reported as “trace positive” (21, 22). Given its clearly superior clinical value (23–26), Ultra is suggested as the preferred molecular diagnostic for children with suspected pulmonary TB (27). WHO guidelines recommend prioritizing mWRDs—such as Xpert MTB/RIF, Ultra—over smear or culture for initial testing in children with pulmonary TB symptoms, using sputum, gastric aspirates, nasopharyngeal aspirate, or stool (12). For suspected tuberculous meningitis, Xpert and Ultra are the recommended initial test in cerebrospinal fluid (CSF) (12). Where feasible, repeat testing of the same specimen type and/or combined testing of different specimen types (e.g., two stool samples, two nasopharyngeal aspirates, or gastric aspirate plus stool) is recommended to improve diagnostic yield compared with single-sample testing (3, 28). For lymph node TB, Xpert MTB/RIF or Ultra may be used as the initial test on aspirate or biopsy specimens for faster TB confirmation and rifampicin resistance assessment (12). The Xpert/Ultra series has evolved from a pulmonary-specific test into a first-line molecular platform for diverse specimens and TB forms, central to pediatric TB diagnosis and treatment. Beyond laboratory-based deployment, the GeneXpert platform has also been adapted for POC use. The GeneXpert Edge enables Xpert MTB/RIF or Ultra at community/household level. A South African study (29) showed >98% acceptance and >95% valid results, reducing screening-to-result time from >two weeks to the same day. The TB Home Study (30) further showed that combining non-sputum specimens (tongue swabs) with portable molecular platforms increases accessibility for child household contacts, offering proof-of-concept for child-centric non-sputum point of care test (POCT).

#### 3.1.2 Truenat platform

The Truenat platform (including MTB, MTB Plus and MTB-RIF Dx) is a chip-based real-time fluorescent PCR platform utilizing disposable microfluidic chips, a portable micro-PCR analyzer, and the Trueprep AUTO automated extraction instrument. It is battery-operated, requires minimal space and infrastructure, and delivers results in ∼30–60 min per test. Consistent with WHO recommendations for rapid molecular diagnostics as initial tests for tuberculosis, Truenat is classified as a low-complexity, near-POC NAAT (12, 13, 31, 32). Alongside Xpert MTB/RIF and Xpert MTB/RIF Ultra, Truenat MTB/MTB Plus is recommended by WHO as an initial molecular diagnostic test for suspected pulmonary TB in children and adults, including people living with HIV. If Truenat MTB/MTB Plus is positive, MTB-RIF Dx is used as a reflex assay for rifampicin resistance detection (12, 32). The diagnostic Performance of Truenat MTB-RIF Dx in Pediatric Populations is shown in Supplementary Table S2. Truenat demonstrates diagnostic efficacy comparable to laboratory-based Xpert, while offering substantially greater decentralization and lower infrastructure requirements, supporting its role as an initial diagnostic test in resource-limited pediatric settings.

However, an indeterminate rifampicin result with MTB positivity, or high clinical suspicion of multidrug-resistant tuberculosis (MDR-TB), still requires supplementary culture or other molecular methods for resistance confirmation. With future publication of pediatric MDR/XDR-TB (extensively drug-resistant tuberculosis) cohort validations and next-generation chips (e.g., MTB-INH), the low-complexity Truenat NAAT platform is expected to better enable individualized management and early detection of pediatric TB in primary care.

#### 3.1.3 Low-complexity NAAT: TB-LAMP

TB-LAMP requires no expensive thermal cyclers or sophisticated equipment, enabling use in primary-level laboratories (33). In adult with pulmonary TB, its sensitivity (74%–78%) and specificity (98%–99%) relative to culture are comparable to Xpert MTB/RIF (34). The 2021 updated guidelines categorized TB-LAMP as a low-complexity manual NAAT (LC-mNAAT) for rapid diagnosis, maintaining its specific recommendation for adult with pulmonary TB (35). Its diagnostic performance in children is shown in Supplementary Table S3. Notably, current evidence is insufficient to support its use as the preferred initial molecular screen. In summary, TB-LAMP offers a practical alternative to enhance microbiological confirmation of children with TB in resource-limited settings where Xpert/Truenat are unavailable. However, its lack of drug-resistance detection limits utility in children at high risk for drug-resistant TB.

#### 3.1.4 Line probe assay (LPA): a key component of drug resistance testing in children

LPA is a molecular drug-resistance test that combines PCR amplification with reverse hybridization. It identifies M.tb and detects mutations linked to resistance to rifampicin, isoniazid, and some second-line drugs within two days. WHO classifies LPAs (e.g., GenoType MTBDRplus for RIF/INH, MTBDRsl for fluoroquinolones/second-line injectables) as WRDs for drug-resistant TB, mainly for reference/regional laboratories with appropriate capacity (12, 36). Sensitivity and specificity of LPA are shown in Supplementary Table S4. Although LPA shows high sensitivity and specificity for RIF/INH resistance detection in smear/culture-positive specimens, its use is limited by the need for relatively complex laboratory infrastructure and technical training. Evidence for its direct application in smear-negative young children with limited specimen volumes is insufficient. Thus, in algorithms of pediatric TB diagnostics, LPA is better suited for subsequent drug-resistance profiling when rifampicin resistance is identified or highly suspected by initial molecular screening tests, rather than as a universal preferred initial screening tool (37).

#### 3.1.5 CRISPR diagnostics: rapid and sensitive emerging technology

CRISPR-Cas-based TB diagnostics utilize Cas12a/Cas13a to recognize M.tb-specific nucleic acids and amplify signal via collateral cleavage, completing assays within two hours (38). Adult studies with sputum and sterile body fluids show many platforms achieve LODs of a few copies/μL, with clinical sensitivity/specificity approaching or exceeding conventional NAATs. Their simple workflows and flexible readouts support potential translation to pediatric settings (31, 39, 40). In children, prospective clinical data on CRISPR-based diagnostics currently derive mainly from two categories of non-sputum specimens: plasma Mtb-cfDNA detection and non-invasive specimens (Supplementary Table S5). Currently, WHO has not included any CRISPR assays in routine TB diagnostic recommendations; CRISPR-TB is in early clinical translation, with particularly limited evidence in children. However, in contrast to cartridge-based or low-complexity NAATs (e.g., Xpert Ultra and Truenat), most CRISPR-TB platforms still require relatively advanced laboratory capacity, including reliable nucleic acid extraction, a pre-amplification step, strict contamination control, and trained molecular personnel. In addition, assay workflows are often multi-step and manually intensive, with limited integration into closed, automated systems, posing challenges for quality assurance, biosafety, and scalability in high-burden, resource-limited settings (39, 41). These requirements currently constrain widespread decentralization and limit routine implementation at primary- or secondary-care levels, particularly in pediatric TB programs where laboratory infrastructure is frequently limited. In the short term, the realistic role of CRISPR-based TB diagnostics is therefore as an emerging supplement to first-line NAATs (e.g., Xpert Ultra/Truenat) in reference laboratories or research settings. Priority should be given to multi-center-prospective studies on non-invasive/minimally invasive specimens (e.g., serum Mtb-cfDNA, stool, tongue/pharyngeal swabs) to validate its incremental value in real-world pediatric scenarios.

#### 3.1.6 Targeted next-generation sequencing (tNGS)/whole-genome sequencing (WGS): the future direction of precision drug resistance diagnosis in children

tNGS and WGS represent a major advance in TB molecular diagnostics, shifting from single-gene testing to comprehensive drug-resistance profiling. In 2024, WHO included tNGS in recommendations for drug susceptibility testing (DST) in confirmed TB (including rifampicin-resistant tuberculosis, i.e., RR-TB) (12). It can simultaneously analyze mutations for multiple first- and second-line drugs on clinical specimens like sputum, serving as an alternative or complement to phenotypic DST in updated algorithms (Supplementary Table S6).

Despite broad prospects in DST prediction and surveillance, WGS/tNGS use is constrained by instrument costs, reagent expenses, and bioinformatics needs, limiting implementation to high-income countries or reference labs. For routine pediatric TB care in most high-burden regions, their short-term role remains as an advanced reference tool for complex cases and resistance management, not first-line screening (3, 42).

### 3.2 Advances in host response and biomarker diagnostics

Due to the paucibacillary nature and difficulty in obtaining sputum specimens in pediatric TB, extensive efforts have been made toward host response-based non-sputum diagnostic tools. These assays are primarily intended for triage and risk stratification and remain at an early stage of pediatric validation (43).

#### 3.2.1 Blood transcriptional signatures and Xpert MTB-host response (MTB-HR)

Multi-omics studies have identified reproducible transcriptomic changes in the peripheral blood of patients with TB, particularly upregulated interferon signaling-related genes, which can distinguish active TB from latent infection or other diseases. For example, the Cepheid Xpert MTB-HR is a blood-based POCT (∼60 min, using finger-prick blood) integrating a three-gene host response signature (GBP5, DUSP3, KLF2) onto a cartridge compatible with the Xpert platform (44). In adult cohorts, MTB-HR has demonstrated diagnostic performance approaching WHO triage target product profile (TTP) thresholds, with reported sensitivities of approximately 84%–90% and specificities of 74%–88% depending on the population and reference standard (45, 46). The RaPaed-TB multicenter cohort (47) enrolled 784 children under 15 years with suspected TB. MTB-HR demonstrated 59.8% sensitivity (95% CI 50.8–68.4) and 90.3% specificity (95% CI 85.5–94.0) against culture-confirmed TB. Sensitivity decreased to 41.6% (95% CI 34.7–48.7) for any microbiologically confirmed TB and to 29.6% (95% CI 25.4–34.2) for a composite clinical reference standard (including highly probable unconfirmed TB), with specificity remaining similar.

Blood transcriptomic signatures, particularly MTB-HR, have advanced from high-dimensional RNA signatures to POC finger-prick testing. In adults, performance approaches the lower threshold of WHO's triage TPP, but sensitivity in children requires further validation.

#### 3.2.2 Host protein biomarkers and multi-protein biosignatures

More mature assay platforms are available for evaluating protein-based biomarkers and are less costly compared with mRNA assays, presenting a more feasible pathway towards scalable pediatric diagnostics. C-reactive protein (CRP) is one of the most extensively studied host protein biomarkers, while a cohort study of 332 children with suspected pulmonary TB (48) showed that, using culture-confirmed TB as the gold standard, CRP at a 10 mg/L threshold achieved only 50.0% sensitivity, 63.3% specificity, and an Area Under the Curve (AUC) of 0.59—well below WHO triage TPP requirements.

To improve diagnostic performance, multi-protein/chemokine biosignatures have been explored. A baseline chemokine signature combining CCL1, CXCL1, and CXCL10 (e.g., via combiROC) achieved >90% sensitivity and specificity for distinguishing active TB from unlikely TB in children (49). Moreover, these biosignatures significantly decreased post-treatment, indicating their potential for both diagnosis and treatment response monitoring (49). Kumar et al. (50) evaluated a plasma matrix metalloproteinase (MMP) profile for pediatric TB, finding a three-MMP signature (MMP-1, MMP-2, MMP-9) achieved 100% sensitivity and specificity for distinguishing active TB from controls. However, these findings are limited by small sample size, insufficient validation, and high overfitting risk. Fossati et al. (51) enrolled 511 children with suspected pulmonary tuberculosis (PTB) across four countries. Using plasma DIA-PASEF proteomics and machine learning, they developed four refined biomarker panels (three–six proteins each). In a held-out test set comparing “confirmed TB” vs. “unlikely TB,” these panels achieved AUCs of 0.87–0.88, meeting WHO's minimum TPP threshold for TB screening/triage tests (≥70% specificity at 90% sensitivity).

From an implementation perspective, host protein–based biomarkers differ substantially in readiness for use in high-burden pediatric settings. Single-analyte assays such as CRP—and pathogen-derived protein markers such as urine LAM are currently the most deployable options, owing to their low cost, rapid turnaround time, and minimal infrastructure requirements; however, their limited diagnostic accuracy—particularly in children without HIV—restricts their role largely to triage or adjunctive decision-making rather than definitive diagnosis (48, 52). In contrast, multi-protein and proteomic biosignatures demonstrate markedly improved diagnostic performance and may meet WHO target product profile benchmarks for screening or triage tests, but their reliance on complex laboratory platforms, advanced analytics, and external validation cohorts currently limits scalability and routine implementation in most high-burden settings (51). Further simplification and validation are required to translate these promising signatures into deployable tools for decentralized pediatric TB care.

Despite their promising diagnostic performance, most multi-protein or chemokine biosignatures currently lack plug-and-play assay formats suitable for routine clinical deployment. Unlike CRP, which can be measured at the bedside using simple, low-cost assays, these biosignatures typically rely on centralized laboratory workflows, including multiplex immunoassays, mass spectrometry–based proteomics, or machine-learning–assisted data processing. As a result, their implementation currently requires substantial laboratory infrastructure, technical expertise, and quality-control capacity, limiting near-term feasibility in primary care and high-burden pediatric settings.

#### 3.2.3 T-cell activation marker TB (TAM-TB): T-cell activation marker-based assa**y**

The TAM-TB assay uses flow cytometry to detect activation/maturation markers (e.g., CD38, HLA-DR, Ki-67, CD27) on Mtb-specific CD4^+^ T cells, enabling differentiation of active TB from tuberculosis infection (TBI)/other diseases. A prospective Tanzanian pediatric study (53) of 290 children with suspected TB found the CD27-based TAM-TB algorithm achieved 83.3% sensitivity (95% CI 58.6–96.4) and 96.8% specificity (95% CI 89.0–99.6) for patients with culture-confirmed active TB vs. patients without TB (results in 24 h). Subsequent adult studies simplified it to a CD38-based assay (one mL whole blood), achieving 82.2% sensitivity, 93.4% specificity, and AUC 0.87 (95% CI 0.84–0.91) for active TB, with comparable performance in individuals living with HIV (54). Thus, TAM-TB is promising for non-sputum TB diagnosis but is currently limited by reliance on flow cytometry, sample freshness requirements, and cost, hindering near-term primary care implementation (31).

#### 3.2.4 Lipoarabinomannan (LAM)-pathogen-derived biomarker

LAM is an M.tb cell-wall glycolipid detectable in urine that offers a non-invasive option for young children unable to provide sputum. WHO recommends AlereLAM only for children living with HIV as well as with suspected TB and strictly as an adjunct test, requiring confirmation alongside clinical assessment, imaging and molecular evidence (52). Next-generation FujiLAM shows superior sensitivity (42%–63%) and specificity (84%–93%) compared with earlier LAM assays, particularly in children living with HIV (55, 56), although performance in people without HIV remains suboptimal.

Current guidance therefore favors integrating urine LAM into multi-component diagnostic pathways rather than using it as a standalone test, especially in resource-limited, high-burden pediatric settings. Future research should enhance sensitivity, reduce batch-to-batch variability, and explore combinations with host biomarkers or molecular tests to develop an affordable, accessible, non-invasive first-line diagnostic tool for children in resource-limited, high-burden settings.

### 3.3 Advances in AI for pediatric tuberculosis screening and diagnostic support

AI has emerged as a promising tool for tuberculosis screening and triage, with chest x-ray–based computer-aided detection (CAD) representing the most clinically advanced application to date. Although WHO recommends deep learning–based CAD systems for adult TB screening, these tools are not currently recommended for pediatric TB diagnosis and are intended to support, rather than replace, clinical assessment and human image interpretation. Pediatric research remains limited, as most existing CAD tools are trained on adult radiographs and do not fully capture children's unique thoracic anatomy and radiographic patterns. Consequently, large, well-annotated pediatric chest x-ray datasets and child-specific CAD systems are urgently needed (57).

Palmer et al. (58) tested the commercial CAD4TB system in children with suspected TB. The unmodified model had an AUC of ∼0.58 for TB-suggestive vs. non-TB radiographs; after fine-tuning, it improved to 0.72. For microbiologically confirmed TB, AUC rose from ∼0.68 to 0.78, demonstrating that adult CAD algorithms are not directly transferable but pediatric-specific training data significantly enhances performance. However, retrospective multinational analyses reveal even optimized systems are inadequate: CAD4TBv7 achieved only 43%–50% sensitivity for confirmed pediatric TB, below screening thresholds (59). Recently, specialized pediatric deep learning systems have emerged. Capellán-Martín et al. (60) developed pTBLightNet using self-supervised and transfer learning on >110,000 adult chest x-rays, then fine-tuned on 918 pediatric patients. Internal-test AUC reached 0.903 for TB-compatible CXR, but external dataset performance dropped to ∼0.68, indicating limited real-world generalizability. To address heterogeneity in reported performance across CAD platforms, the Stop TB Partnership and FIND have established a public CAD platform comparator that enables standardized, head-to-head evaluation of multiple commercial and non-commercial CAD systems using common reference datasets, supporting transparent benchmarking and evidence generation for policy guidance (61). Beyond radiography, AI integrates multimodal data. In a prospective cohort of children <five with suspected TB, Smith et al. (62) trained 11 models combining clinical, exposure, immunologic, and imaging features to predict microbiologically positive samples. AUROCs were 0.84–0.90 for invasive specimens and 0.83–0.89 for non-invasive ones, showing AI's potential to target high-yield sampling and improve confirmation. Machine learning also screens novel M.tb antigen-cytokine combinations for diagnostics (63).

In summary, AI in pediatric TB diagnosis has advanced from applying CAD algorithms trained on adult radiographs to developing algorithms and multimodal frameworks based on pediatric radiographs. However, most studies are retrospective, use small samples, and under-represent high-risk subgroups. Future development requires large, standardized pediatric datasets and real-world trials to confirm if AI reduces diagnostic delays or improves outcomes across settings.

### 3.4 Advances in immunodiagnostics of TBI

Immunodiagnostic tools, primarily Interferon-Gamma Release Assays (IGRAs) and the Tuberculin Skin Test (TST) serve as adjunctive tests for PTB, yet they continue to play an essential role in clinical decision-making, especially when microbiologic confirmation is challenging in children. Recent years have seen a notable increase in evidence for immunodiagnostics of TBI in children, particularly concerning the real-world performance of IGRAs (including T-SPOT.TB and QuantiFERON) and the application of novel TSTs. A systematic review (64) of 9,065 children reported that IGRAs achieved a pooled sensitivity of 0.85 (95% CI: 0.79–0.89) and specificity of 0.94 (95% CI: 0.88–0.97) when evaluated against microbiological reference standards in cohorts of children with suspected active TB, reflecting their performance as adjunctive tests rather than stand-alone diagnostics for TB disease. However, sensitivity dropped to 0.77–0.85 in children under five years old, indicating reduced performance in younger children and reinforcing the need for cautious interpretation in this population. QuantiFERON-TB Gold Plus (QFT-Plus) demonstrates overall stable performance, but its sensitivity significantly decreases in children with central nervous system tuberculosis, disseminated TB, and those who are immunocompromised (65, 66).

LIOFeron® TB/LTBI, a new IGRA platform, demonstrated higher sensitivity (96.8% vs. 89.3%) and AUC (0.997 vs. 0.922) than QFT-Plus in an Italian single-center pediatric study (67). QIAreach QuantiFERON-TB, a near-POC version of QFT-Plus with a single-use chip and lateral flow readout (20-minute interpretation, smaller blood volume), performed well in adults (68), but showed poor agreement with QFT-Plus (*κ* = 0.13) and unstable results in children aged one to four years; production has been suspended.

TST has limited specificity in high- Bacillus Calmette-Guérin (BCG)-vaccination settings due to cross-reactivity with BCG and nontuberculous mycobacteria (NTM). Novel ESAT-6/CFP-10–based TBSTs improve specificity: a Russian study of 2,710 children reported Diaskintest (DT) positivity of 11.7% vs. 63.1% for TST (≥10 mm), with poor agreement (*κ* = 0.08), indicating DT reduces false positives (69). A Phase III trial of C-TST in China showed higher sensitivity than TST and T-SPOT, with good safety in children (70).

### 3.5 Comparative advantages and limitations

Across molecular and biomarker-based diagnostics, POC and near-POC deployability—defined as patient-proximate testing with rapid turnaround (typically ≤60–90 min) and minimal infrastructure or operator requirements—represents a critical determinant of clinical impact in pediatric TB, particularly in high-burden, resource-limited settings (71). Although WHO-recommended molecular diagnostics share the goal of improving microbiological confirmation in children, each test type presents distinct advantages and constraints. Cartridge-based assays (Xpert MTB/RIF and Ultra) offer highly standardized, automated workflows with integrated rifampicin resistance detection, supporting consistent implementation and broad specimen compatibility; however, they require stable electricity, controlled laboratory environments, and higher per-test costs, limiting decentralization (12). Portable configurations of the GeneXpert platform (e.g., GeneXpert Edge) partially mitigate these constraints and enable community- or household-level deployment but still depend on cartridge supply chains and platform maintenance. Ultra improves sensitivity in paucibacillary and non-invasive specimens but introduces reduced specificity due to trace-positive results (21, 22). Truenat provides substantially lower infrastructure and power requirements and enables near-POC deployment but relies on reflex testing for resistance confirmation and currently has more limited pediatric validation for second-line resistance detection (12, 32). TB-LAMP offers minimal equipment needs but lacks drug-resistance detection and shows variable, specimen-dependent performance in children (35). Line probe assays and sequencing-based approaches provide high-resolution resistance profiling but remain constrained to reference laboratories due to infrastructure, biosafety, and technical demands (12, 36, 42).

Beyond these established platforms, emerging near-POC molecular tests such as the PlusLife MiniDock MTB Test (Guangzhou Pluslife Biotech) have recently been evaluated in multiple outpatient and community-based settings. Adult studies using tongue swabs and sputum specimens reported diagnostic sensitivity and specificity comparable to existing near-POC NAATs, including Xpert MTB/RIF Ultra, and broadly consistent with World Health Organization target product profile benchmarks for non-sputum-based TB diagnostics (72, 73). However, clinical validation in pediatric populations remains limited or absent, and the MiniDock platform does not provide rifampicin resistance detection, which currently restricts its role in comprehensive pediatric TB diagnostic algorithms.

## 4 Discussion

Pediatric tuberculosis is entering a pivotal era: after decades of reliance on adult evidence, an increasingly child-specific data foundation is emerging most notably in pediatric diagnostic technologies. Yet, the central challenge remains the persistent divide between scientific innovation and programmatic implementation—particularly in high-burden, resource-limited settings where the disease burden is most concentrated.

Despite meaningful diagnostic advances, no currently available test fully satisfies WHO TPPs for a child-friendly triage or rule-out test. Vulnerable subgroups—including infants, children living with HIV, children with malnutrition, and those with extrapulmonary TB—remain the most underserved. While molecular platforms have enhanced confirmation capacity, and newer modalities (e.g., urine LAM, host-response biomarkers, CRISPR-based assays, and AI-assisted imaging) hold promise for non-sputum detection, most lack sufficient prospective, multi-center pediatric validation and remain largely confined to research or reference-laboratory settings. Accordingly, there is a critical lack of evidence demonstrating tangible clinical impact, such as reduced diagnostic delay, improved linkage to treatment, or mortality. Future priorities must therefore extend beyond analytical performance benchmarks toward implementation science, including rigorous evaluation of non-sputum tools (e.g., stool, urine, tongue swabs), co-development of platforms with decentralized delivery systems; and robust cost-effectiveness analyses to drive scalable adoption.

Collectively, these gaps underscore a structural challenge: advances remain siloed, whereas children experience TB as a continuous journey from exposure to diagnosis and care. Future success will depend on strategies that bridge evidence generation and policy implementation, with a deliberate focus on the most diagnostically complex and vulnerable pediatric populations. For the coming decade, priorities should center on validated non-sputum diagnostic tools deployable at or near the point of care, integrated diagnostic algorithms suited to primary health systems, and implementation strategies that ensure equitable access in high-burden settings. Achieving this requires not just the incremental adaptation of adult tools, but a reimagined pediatric TB diagnostic paradigm designed explicitly around the biological, clinical, and programmatic realities of children.
