# Supplementary material for: From gaps to progress: five-year global advances in the diagnosis of pediatric tuberculosis
Source: Front Pediatr. 2026 Mar 17;14:1778870. doi: 10.3389/fped.2026.1778870 (PMC13036138; doi:10.3389/fped.2026.1778870)
Supplement: Supplementary file 1 [file Supplementaryfile1.docx]

FigureS1: PRISMA-style (PRISMA-informed) flow diagram.


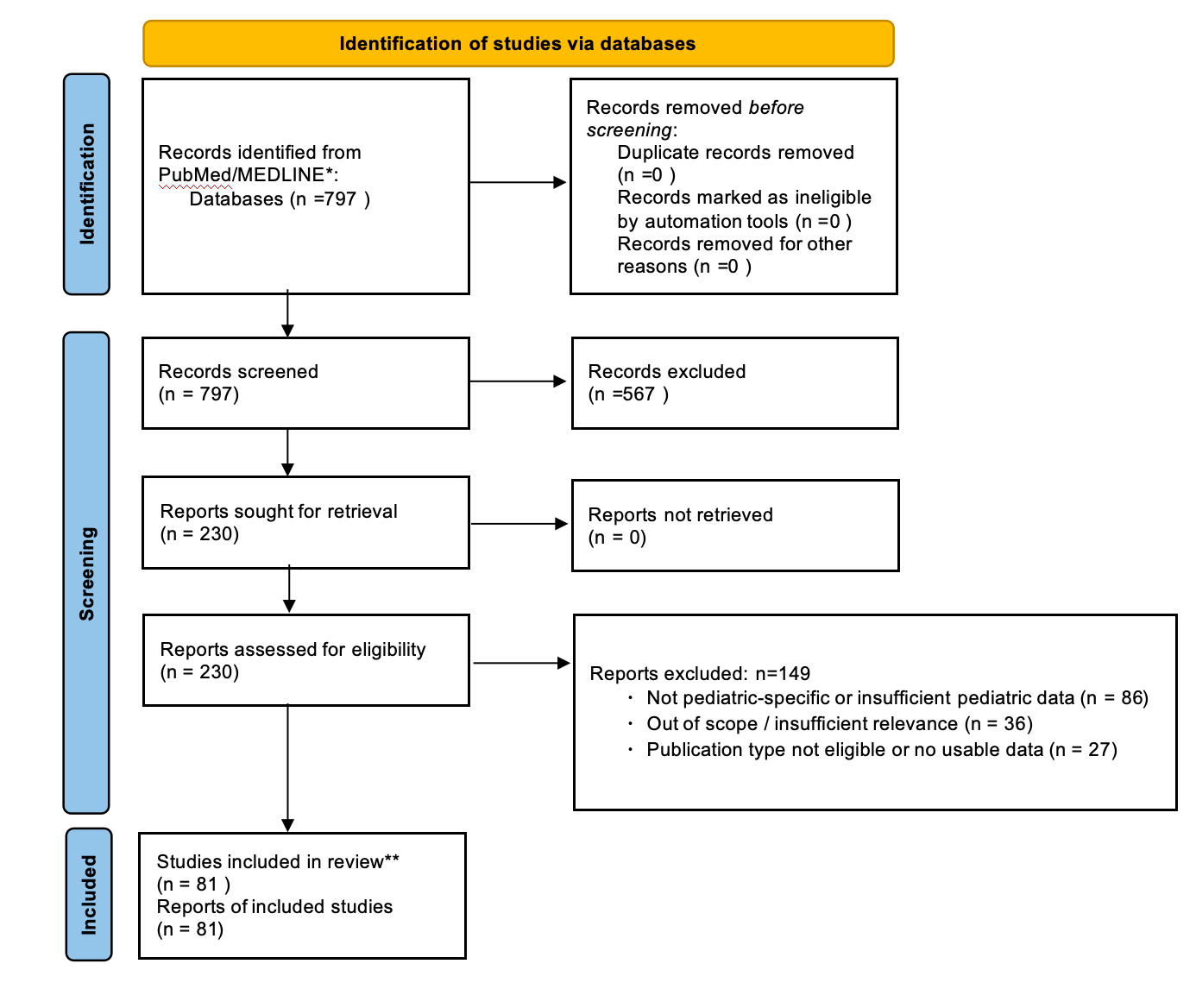
*Additional records were identified from Web of Science (n=1689), Embase (n=1165), Scopus (n=2000), Cochrane Library (n=318), Google Scholar, guideline repositories, and reference-list screening; these sources informed inclusion but were not subjected to independent quantitative screening counts.

**Additional references (e.g., WHO reports, policy documents, and seminal background studies) were cited to provide epidemiologic context and were not counted as included studies in the PRISMA flow diagram.

Table S1: Key Diagnostic Performance of Xpert MTB/RIF and Xpert Ultra in Children

| Parameter | Xpert MTB/RIF | Xpert MTB/RIF Ultra | Source |
| --- | --- | --- | --- |
| Function | Detects MTB + rifampicin resistance | Next-generation MTB NAAT improving sensitivity | [1, 2] |
| Sensitivity – sputum | 64.6% (95% CI 55.3–72.9%)^#^ | 74.3% (single induced sputum) ^*^; ~80% (with induced sputum + NPA) | [3, 4] |
| Sensitivity – GA | 73.0% (95% CI 52.9–86.7%)^#^ | 69.6% (95% CI: 60.3–77.6%）^*^ | [3-8] |
| Sensitivity – stool | 61.5% (95% CI 44.1–76.4%)^#^ | 58.6% (vs. 37.9% MTB/RIF) using induced sputum culture as reference | [3, 6] |
| Sensitivity – nasopharyngeal specimen | 45.7% (95% CI 27.6–65.1%)^#^ | 43.7% (95% CI: 26.7–62.2%) ^#^ | [3, 4, 9] |
| Specificity – Above sample types | >98% across specimens^#^ | 94.1%~97.5% across specimens^#^ | [3, 9]，[10, 11] |
| Rifampicin-resistance detection | Sensitivity 90.0% (95% CI 67.6–97.5%); specificity 98.3% (95% CI 87.7–99.8%) | NA | [3, 9] |
| Overall recommendation | Practical, valuable initial test especially where DS-TB evaluation is needed | WHO-preferred initial mWRD; applicable to stool, GA, and NPA | [9, 12] |

# against M.tb culture, *against microbiologically confirmed TB, DS-TB: drug-susceptible tuberculosis, GA: gastric aspirate, NPA: nasopharyngeal aspirate.

Table S2. Diagnostic Performance of Truenat MTB-RIF Dx

| Study / Setting | Reference Standard | Sensitivity | Specificity | Key Notes |
| --- | --- | --- | --- | --- |
| Multicenter pediatric study (GA, induced sputum, BALF) [13] | MRS | 57.1% | 92.0% | Comparable to GeneXpert MTB/RIF (56.0% / 91.4%) |
| Same multi-center study [13] | MGIT liquid culture | 58.7% | 87.5% | — |
| Adult-dominant pooled analyses [14] | CRS | ~91.0% | ~96.0% | Illustrates gap between Adult reference |

GA: gastric aspirate, BALF: bronchoalveolar lavage fluid, MRS: microbiological reference standard, CRS: composite reference standard.

Table S3: Diagnostic Performance of TB-LAMP in Pediatric Tuberculosis

| Study | Population & Specimens | Reference Standard | Sensitivity | Specificity | Key Notes |
| --- | --- | --- | --- | --- | --- |
| Cochrane Review (303 children <10 years) [15] | Respiratory specimens; GA/lavage; NPA | Culture | 50–100% | 67–100% | High specimen-dependent variability; insufficient evidence as first-line test. |
| India cross-sectional pediatric study (n=60) [16] | Suspected PTB | Culture | 75.0% | 72.4% | Comparable to PCR; superior to smear microscopy |
| China BALF pediatric cohort (n=243) [17] | Children with bacteriologically confirmed PTB | Culture / CRS | 77.5% | — | ~40% added yield in smear/culture-negative disease; useful in paucibacillary cases. |

GA: gastric aspirate, NPA: nasopharyngeal aspirate, PTB: pulmonary tuberculosis; CRS: Composite Reference Standard.

Table S4: Diagnostic Utility of Line Probe Assays (LPA)

| Study | Population & Specimen | Reference Standard | Diagnostic Performance | Key Takeaways |
| --- | --- | --- | --- | --- |
| Adult pooled meta-analysis (benchmark) [18] | PTB adults; sputum | Culture / phenotypic DST | •RIF-mono: Sens/ Spec: 96%/98%  •INH-mono: Sens/ Spec: 91%/99%  •MDR-TB: Sens/ Spec: 91%/99% | Accurate, rapid (1–2-day) resistance screening. |
| Nigeria pediatric cohort [19] | Suspected pediatric PTB; GA, sputum | — | •Overall LPA positivity: 45.6%  •GA/smear  positivity: 41.8%/3.0%  •Sputum: 58.1% vs 16.1% smear | Higher yield than smear in paucibacillary pediatric TB; supports pediatric MDR-TB programs |
| Pediatric MDR-TB program data [20], [21, 22] | MDR-TB children; respiratory specimens | CRS | Qualitative evidence only – consistently higher sensitivity than smear; high specificity reported when smear-positive | Supports mutation profiling; pediatric sensitivity data limited. |

GA: gastric aspirate, DST: drug susceptibility testing, CRS: composite reference standard, Sens: sensitivity, Spec: specificit

Table S5: Evidence Summary of CRISPR-Cas TB Diagnostics in Pediatric Populations

| Study | Population & Specimen | Reference Standard | Key Performance | Key Implication |
| --- | --- | --- | --- | --- |
| Huang et al., Eswatini[23] | Suspected pediatric TB; cfDNA | CRS | Sens/Spec: 83%/95% | Strong signal in paucibacillary and extrapulmonary TB |
| Huang et al., Kenya[23] | HIV-positive children; cfDNA | MRS / clinical classification | MRS: Sens: 100%  Clinical TB: Sens: 85% | cfDNA decreases with treatment → potential monitoring role |
| Huang et al., 2025[24] | Suspected pediatric PTB; Stool | Induced sputum culture | Sens/Spec:83%/94% | First proof-of-concept for non-invasive stool-based screening |

CRS: composite microbiological reference, MRS: microbiological reference standard, Sens: sensitivity, Spec: specificity

Table S6: Clinical and Programmatic Evidence for tNGS and WGS in Tuberculosis

| Category | Citation | Population & Specimen | Reference Standard | Diagnostic Performance | Clinical Role |
| --- | --- | --- | --- | --- | --- |
| tNGS | Pooled evidence[25]. | Mostly adults; mixed clinical specimens (incl. pediatric extrapolation) | Phenotypic DST | Sensitivity: 94.1% • Specificity: 98.1% | Comprehensive genotypic DST for 1st/2rd-line drugs; WHO-endorsed |
| tNGS | China cohort [26] | Suspected TB (n=313; 85 children); respiratory & extrapulmonary samples) | CRS | Sensitivity: 74% Specificity: 97% | Single-specimen TB detection and resistance profiling; useful for pediatric extrapulmonary TB |
| tNGS | Pediatric subset [27] | Suspected pediatric PTB; respiratory samples | Clinical diagnosis | Sensitivity: 18.4% Specificity: 87.1% | Complementary to Xpert in culture(-)/suspected drug-resistant/retreatment cases |
| WGS | Surveillance studies [28, 29]. | Adults; sequenced isolates (population-level) | NA | NA (epidemiologic application) | High-resolution transmission and resistance surveillance; not for frontline diagnosis |

DST: drug susceptibility testing, CRS: composite reference standard, NA: Not applicable.

**REFERENCES**

1. Nurwidya F, Handayani D, Burhan E, Yunus F. Molecular diagnosis of tuberculosis. Chonnam Med J. 2018;54(1):1-9.
2. Kohli M, Schiller I, Dendukuri N, Yao M, Dheda K, Denkinger CM, et al. Xpert MTB/RIF Ultra and Xpert MTB/RIF assays for extrapulmonary tuberculosis and rifampicin resistance in adults. Cochrane Database Syst Rev. 2021;1(1):CD012768.
3. Kay AW, Gonzalez Fernandez L, Takwoingi Y, Eisenhut M, Detjen AK, Steingart KR, et al. Xpert MTB/RIF and Xpert MTB/RIF Ultra assays for active tuberculosis and rifampicin resistance in children. Cochrane Database Syst Rev. 2020;8(8):CD013359.
4. Zar HJ, Workman LJ, Prins M, Bateman LJ, Mbhele SP, Whitman CB, et al. Tuberculosis diagnosis in children using Xpert Ultra on different respiratory specimens. Am J Respir Crit Care Med. 2019;200(12):1531-8.
5. Nicol MP, Workman L, Prins M, Bateman L, Ghebrekristos Y, Mbhele S, et al. Accuracy of Xpert MTB/RIF Ultra for the diagnosis of pulmonary tuberculosis in children. Pediatr Infect Dis J. 2018;37(10):e261-3.
6. Kabir S, Rahman SMM, Ahmed S, Islam MS, Banu RS, Shewade HD, et al. Xpert Ultra assay on stool to diagnose pulmonary tuberculosis in children. Clin Infect Dis. 2021;73(2):226-34.
7. Kay AW, Madison M, Scandrett K, Ness T, Amuge P, Inbaraj LR, et al. Xpert MTB/RIF Ultra assay for tuberculosis disease and rifampicin resistance in children. Cochrane Database Syst Rev. 2025;10(10):CD013359.
8. Carratala-Castro L, Munguambe S, Saavedra-Cervera B, de Haas P, Kay A, Marcy O, et al. Performance of stool-based molecular tests for paediatric tuberculosis diagnosis: a systematic review and meta-analysis. Lancet Microbe. 2025;6(6):100963.
9. Kay AW, Ness T, Verkuijl SE, Viney K, Brands A, Masini T, et al. Xpert MTB/RIF Ultra assay for tuberculosis disease and rifampicin resistance in children. Cochrane Database Syst Rev. 2022;9(9):CD013359.
10. Bouzouita I, Ghariani A, Dhaou KB, Jemaeil S, Essaalah L, Bejaoui S, et al. Usefulness of Xpert MTB/RIF Ultra for rapid diagnosis of extrapulmonary tuberculosis in Tunisia. Sci Rep. 2024;14(1):2217.
11. Slail MJ, Booq RY, Al-Ahmad IH, Alharbi AA, Alharbi SF, Alotaibi MZ, et al. Evaluation of Xpert MTB/RIF Ultra for extrapulmonary tuberculosis: a retrospective analysis in Saudi Arabia. J Epidemiol Glob Health. 2023;13(4):782-93.
12. WHO consolidated guidelines on tuberculosis. Module 3: diagnosis – rapid diagnostics for tuberculosis detection. 3rd ed. Geneva: World Health Organization; 2024.
13. Singh UB, Singh M, Sharma S, Mahajan N, Bala K, Srivastav A, et al. Expedited diagnosis of pediatric tuberculosis using Truenat MTB-Rif Dx and GeneXpert MTB/RIF. Sci Rep. 2023;13(1):6976.
14. Inbaraj LR, Daniel J, Sathya Narayanan MK, Srinivasalu VA, Bhaskar A, Scandrett K, et al. Truenat MTB assays for pulmonary tuberculosis and rifampicin resistance in adults and adolescents. Cochrane Database Syst Rev. 2025;3(3):CD015543.
15. Inbaraj LR, Sathya Narayanan MK, Daniel J, Srinivasalu VA, Bhaskar A, Daniel BD, et al. Low-complexity manual nucleic acid amplification tests for pulmonary tuberculosis in children. Cochrane Database Syst Rev. 2025;6(6):CD015806.
16. Sreedeep KS, Sethi S, Yadav R, Vaidya PC, Angurana SK, Saini A, et al. Loop-mediated isothermal amplification in respiratory specimens for pediatric pulmonary tuberculosis: a pilot study. J Infect Chemother. 2020;26(8):823-30.
17. Fan L, Guan B, Cheng M, Liu C, Tian Y, Li R, et al. Evaluation of a loop-mediated isothermal amplification assay for pulmonary tuberculosis in children using bronchoalveolar lavage fluid. Infect Drug Resist. 2022;15:975-87.
18. Bai Y, Wang Y, Shao C, Hao Y, Jin Y. GenoType MTBDRplus assay for rapid detection of multidrug resistance in Mycobacterium tuberculosis: a meta-analysis. PLoS One. 2016;11(3):e0150321.
19. Ebonyi AO, Oguche S, Abok II, Isa YO, Ani CC, Akhiwu HO, et al. Improving the diagnosis of pulmonary tuberculosis using line probe assay and determining factors associated with disease in children in Jos, Nigeria. Germs. 2020;10(4):328-37.
20. Arora J, Singhal R, Bhalla M, Verma A, Singh N, Behera D, et al. Drug resistance detection and mutation patterns of multidrug-resistant tuberculosis strains from children in Delhi. J Epidemiol Glob Health. 2017;7(2):141-5.
21. Bangarwa M, Dhingra D, Mittal M, Saigal K, Ghosh A. Role of line probe assay in detection of Mycobacterium tuberculosis in children with pulmonary tuberculosis. Indian J Tuberc. 2023;70(Suppl 1):S100-3.
22. Rajendran P, Murugesan B, Balaji S, Shanmugam S, Palanisamy S, Ramamoorthy T, et al. Standardization of a stool concentration method for Mycobacterium tuberculosis detection in the pediatric population. Int J Mycobacteriol. 2022;11(4):371-7.
23. Huang Z, LaCourse SM, Kay AW, Stern J, Escudero JN, Youngquist BM, et al. CRISPR detection of circulating cell-free Mycobacterium tuberculosis DNA in adults and children. Lancet Microbe. 2022;3(7):e482-92.
24. Huang Z, Song Z, Zeng J, Liu X, Fang M, Wu Z, et al. Sensitive pathogen DNA detection by a multi-guide RNA Cas12a assay. Nat Commun. 2025;16(1):8257.
25. Schwab TC, Perrig L, Goller PC, Guebely De la Hoz FF, Lahousse AP, Minder B, et al. Targeted next-generation sequencing to diagnose drug-resistant tuberculosis: a systematic review and meta-analysis. Lancet Infect Dis. 2024;24(10):1162-76.
26. Chen Y, Fan L, Ren Z, Yu Y, Sun J, Wang M, et al. Sensitive diagnosis of paucibacillary tuberculosis with targeted next-generation sequencing. BMC Med. 2025;23(1):178.
27. Zheng H, Yang H, Wang Y, Li F, Xiao J, Guo Y, et al. Diagnostic value of tNGS vs Xpert MTB/RIF in childhood TB. Heliyon. 2024;10(1):e23217.
28. Zhdanova S, Jiao WW, Sinkov V, Khromova P, Solovieva N, Mushkin A, et al. Insight into population structure and drug resistance of pediatric tuberculosis strains from China and Russia gained through whole-genome sequencing. Int J Mol Sci. 2023;24(12):10302.
29. Masiarova S, Dvorakova V, Hromadkova M, Norman A, Kunc P, Fabry J, et al. Genotyping and transmission analysis of Mycobacterium tuberculosis in a pediatric population in Czech Republic and Slovakia. BMC Infect Dis. 2025;25(1):891.
